# Supplementary material for: The quantum-optical nature of high harmonic generation
Source: Nat Commun. 2020 Sep 14;11:4598. doi: 10.1038/s41467-020-18218-w (PMC7490274; doi:10.1038/s41467-020-18218-w)
Supplement: Supplementary file 2 — Supplementary Information [file 41467_2020_18218_MOESM2_ESM.pdf]

The Quantum-Optical Nature of High Harmonic Generation

Gorlach et al.

Supplementary Information

Supplementary Note 1: Decomposition of a fully quantized field into a classical part and a quantum part

Here, we set up the basic problem under consideration: an electronic system (such as an atom) that interacts with an intense laser field and emits radiation (with a high harmonic generation being a key example). We consider both the driving field and the emitted field to be fully quantized. In this section, we show that by performing a suitable unitary transformation generated by a "displacement operator", the fully quantized field can be expressed in terms of the sum of a classical (c-number) driving term and a quantized term. This transformation closely follows the treatment in [1].

The free electromagnetic (EM) field in a vacuum has the following form [1]:

$$\mathbf{A} = \sum_{\mathbf{k}\sigma} \sqrt{\frac{\hbar}{2\varepsilon_0 V c k}} \left[\mathbf{e}_\sigma a_{\mathbf{k}\sigma} e^{i\mathbf{k}\cdot\mathbf{r}} + \mathbf{e}_\sigma^* a_{\mathbf{k}\sigma}^\dagger e^{-i\mathbf{k}\cdot\mathbf{r}}\right]. \tag{1}$$

where V is the volume of space, $\sum_{\mathbf{k}\sigma}$ represents a summation over all possible photonic modes characterized by polarization σ and wavevector $\mathbf{k}$. The operators $a_{\mathbf{k}\sigma}$ and $a_{\mathbf{k}\sigma}^\dagger$ (here † means Hermitian conjugation) are the annihilation and creation operators of a photon, $\mathbf{e}_\sigma$ is a unit vector of polarization, ε_0 is the vacuum permittivity and c is the speed of light.

The interaction between an electron in an external potential U and the electromagnetic field can be described by the following Hamiltonian [1, 2, 3]:

$$H = \frac{1}{2m}(\mathbf{p} - q\mathbf{A})^2 + U + H_{\mathrm{F}}, \tag{2}$$

where $\mathbf{p}$ is the momentum operator, q is the electron charge, m is the electron mass, $H_{\mathrm{F}} = \sum_{\mathbf{k}\sigma} \hbar\omega_k a_{\mathbf{k}\sigma}^\dagger a_{\mathbf{k}\sigma}$ is the Hamiltonian of the electromagnetic field and $\omega_k = ck$ is the frequency of a photon of wavenumber $k = |\mathbf{k}|$. The procedure below works in the exact same way for any number of electrons, since it only involves the photonic parts.

When one probes an electronic system with a laser field, the combined wavefunction of the matter and laser field before their interaction can be written in a factorized form $|\Psi_{\mathrm{i}}\rangle = |\phi_{\mathrm{i}}\rangle|\psi_{\mathrm{laser}}\rangle$, with $|\phi_{\mathrm{i}}\rangle$ the initial state of the electronic system and $|\psi_{\mathrm{laser}}\rangle$ is the quantum state of the laser field,

which is well-described as a multi-mode coherent state of the form $|\psi_{\text{laser}}\rangle = \prod_{\mathbf{k}\sigma} |\alpha_{\mathbf{k}\sigma} \mathrm{e}^{-i\omega_k t}\rangle$. The $\alpha_{\mathbf{k}\sigma}$ are essentially the modal coefficients of the classical electric field, expressed as a sum of all possible plane-waves. In what follows, we will perform a unitary transformation of the Hamiltonian to "transform away" the quantum state of the laser field. The net result of this transformation will be that the coherent state of the driving laser transforms to the vacuum state, and the vector potential operator is shifted by a c-number function corresponding to the classical time-dependent vector potential associated with the laser field. Therefore, the photonic state will become simpler, at the price of the Hamiltonian becoming time-dependent.

The unitary transformation is generated by the "displacement operator" $D(t)$

$$D(t) = \prod_{\mathbf{k}\sigma} \exp\left[\alpha_{\mathbf{k}\sigma} \mathrm{e}^{-i\omega_k t} a_{\mathbf{k}\sigma}^\dagger - \alpha_{\mathbf{k}\sigma}^* \mathrm{e}^{i\omega_k t} a_{\mathbf{k}\sigma}\right]. \tag{3}$$

The displacement operator $D(t)$ has the following properties [4]:

$$\begin{cases} D(t)|0\rangle = \prod_{\mathbf{k}\sigma} |\alpha_{\mathbf{k}\sigma} \mathrm{e}^{-i\omega_k t}\rangle \\ D(t)D^\dagger(t) = 1 \\ D^\dagger(t) a_{\mathbf{k}\sigma} D(t) = a_{\mathbf{k}\sigma} + \alpha_{\mathbf{k}\sigma} \mathrm{e}^{-i\omega_k t} \\ D(t) a_{\mathbf{k}\sigma} D^\dagger(t) = a_{\mathbf{k}\sigma} - \alpha_{\mathbf{k}\sigma} e^{-i\omega_k t} \end{cases}. \tag{4}$$

We may express the wavefunction $|\Psi(\mathrm{t})\rangle$ in terms of a transformed wavefunction $|\widetilde{\Psi}\rangle$ defined such that

$$|\Psi\rangle = D(t)|\widetilde{\Psi}\rangle, \tag{5}$$

with $|\Psi\rangle$ satisfying the Schrodinger equation $i\hbar \frac{\partial |\Psi\rangle}{\partial t} = H|\Psi\rangle$ with the Hamiltonian in Supplementary Eq. (2). Substituting the transformed wavefunction, one immediately has that:

$$i\hbar \frac{\partial |\widetilde{\Psi}\rangle}{\partial t} = D^\dagger H D|\widetilde{\Psi}\rangle - i\hbar D^\dagger \frac{\partial D}{\partial t}|\widetilde{\Psi}\rangle.$$

Using the definition of D in Supplementary Eq. (3), along with properties of the displacement operator (Supplementary Eq. (4)), it can be shown that:

$$i\hbar D^\dagger \frac{\partial D}{\partial t} = \sum_{\mathbf{k}\sigma} \hbar\omega_k \left(\alpha_{\mathbf{k}\sigma} \mathrm{e}^{-i\omega_k t} a_{\mathbf{k}\sigma}^\dagger + \alpha_{\mathbf{k}\sigma}^* \mathrm{e}^{i\omega_k t} a_{\mathbf{k}\sigma} + |\alpha_{\mathbf{k}\sigma}|^2\right),$$

$$D^\dagger H D - i\hbar D^\dagger \frac{\partial D}{\partial t} = \left(\frac{\mathbf{p}^2}{2m} + U\right) - \frac{q}{2m}\mathbf{p} \cdot D^\dagger \mathbf{A} D - \frac{q}{2m} D^\dagger \mathbf{A} D \cdot \mathbf{p} + \frac{q^2}{2m}\left(D^\dagger \mathbf{A} D\right)^2 + \left(D^\dagger H_{\mathrm{F}} D - i\hbar D^\dagger \frac{\partial D}{\partial t}\right),$$

$$\left(D^\dagger H_{\mathrm{F}} D - i\hbar D^\dagger \frac{\partial D}{\partial t}\right) = H_{\mathrm{F}} = \sum_{\mathbf{k}\sigma} \hbar\omega_k a_{\mathbf{k}\sigma}^\dagger a_{\mathbf{k}\sigma}.$$

From the action of the displacement operator on creation and annihilation operators, it immediately follows that:

$$D^{\dagger}\mathbf{A}D = \mathbf{A}_{\mathrm{c}}(t) + \mathbf{A}_{\mathrm{q}},$$

where $\mathbf{A}_{\mathrm{c}}(t) = \langle\psi_{\mathrm{laser}}|\mathbf{A}|\psi_{\mathrm{laser}}\rangle = \sum_{\mathbf{k}\sigma}\sqrt{\frac{\hbar}{2\varepsilon_0 Vck}}\left[\mathbf{e}_{\sigma}\alpha_{\mathbf{k}\sigma}\mathrm{e}^{i\mathbf{k}\cdot\mathbf{r}-i\omega_k t} + \mathbf{e}_{\sigma}^{*}\alpha_{\mathbf{k}\sigma}^{*}e^{-i\mathbf{k}\cdot\mathbf{r}+i\omega_k t}\right]$ and $\mathbf{A}_{\mathrm{q}} = \sum_{k\sigma}\sqrt{\frac{\hbar}{2\varepsilon_0 Vck}}\left[\mathbf{e}_{\sigma}a_{\mathbf{k}\sigma}e^{i\mathbf{k}\cdot\mathbf{r}} + \mathbf{e}_{\sigma}^{*}a_{\mathbf{k}\sigma}^{\dagger}e^{-i\mathbf{k}\cdot\mathbf{r}}\right]$.

It follows that the Hamiltonian transforms according to:

$$D^{\dagger}HD - i\hbar D^{\dagger}\frac{\partial D}{\partial t} = \left[\frac{1}{2m}\left(\mathbf{p} - q\mathbf{A}_{\mathrm{c}}(t)\right)^2 + U\right] - \frac{q}{2m}\left(\mathbf{p} - q\mathbf{A}_{\mathrm{c}}(t)\right)\cdot\mathbf{A}_{\mathrm{q}} - \frac{q}{2m}\mathbf{A}_{\mathrm{q}}\cdot\left(\mathbf{p} - q\mathbf{A}_{\mathrm{c}}(t)\right) + \frac{q^2}{2m}\mathbf{A}_{\mathrm{q}}^2 + H_{\mathrm{F}}.$$

It also follows from the properties of the displacement operator that $|\widetilde{\Psi}_{\mathrm{i}}\rangle = |\phi_{\mathrm{i}}\rangle|0\rangle$. Therefore, the laser coherent state transforms into the vacuum state. Since the typical magnitude of a matrix element of $\mathbf{A}_{\mathrm{q}}$ is on the order of $\sqrt{\frac{\hbar}{2\varepsilon_0 Vck}}$, the typical magnitude of $\frac{q^2}{2m}\mathbf{A}_{\mathrm{q}}^2$ is far smaller than any other energy scale in the problems that we consider, and we will neglect this term henceforth. The right-hand side of the transformed Schrodinger equation is given by

$$D^{\dagger}HD - i\hbar D^{\dagger}\frac{\partial D}{\partial t} = \left[\frac{1}{2m}\left(\mathbf{p} - q\mathbf{A}_{\mathrm{c}}(t)\right)^2 + U\right] + H_{\mathrm{F}} - \frac{q}{2m}\left(\mathbf{p} - q\mathbf{A}_{\mathrm{c}}(t)\right)\cdot\mathbf{A}_{\mathrm{q}} - \frac{q}{2m}\mathbf{A}_{\mathrm{q}}\cdot\left(\mathbf{p} - q\mathbf{A}_{\mathrm{c}}(t)\right).$$

Therefore, the result of the transformation can be summarized as:

$$i\hbar\frac{\partial|\widetilde{\Psi}\rangle}{\partial t} = H|\widetilde{\Psi}\rangle, \tag{6}$$

where

$$H = H_{\mathrm{TDSE}} + H_{\mathrm{F}} + V(t),$$

$$\begin{cases} H_{\mathrm{TDSE}} = \left[\frac{1}{2m}\left(\mathbf{p} - q\mathbf{A}_{\mathrm{c}}(t)\right)^2 + U\right] \\ V(t) = -\frac{q}{2m}\left(\mathbf{A}_{\mathrm{q}}\cdot\left(\mathbf{p} - q\mathbf{A}_{\mathrm{c}}(t)\right)\right) \end{cases},$$

The net result of the transformation enacted in this section is that the effect of the laser field can be taken into account classically (as is typically the case in strong-field physics), while the emission manifests itself as changes in the photon number in the transformed frame. This will be clarified in Supplementary Notes 2-4.

Supplementary Note 2: Radiation emission in strong-field quantum electrodynamics (SFQED)

In this section, we provide a self-contained review of the formulation of time-dependent perturbation theory for systems in which the unperturbed Hamiltonian is time-dependent. As we detail in later sections, we will consider high-harmonic generation as a process of photon emission involving transitions between time-dependent states of the Hamiltonian of an electronic system in a strong time-dependent field. We will use perturbation theory to calculate the corresponding transition probabilities.

We start by introducing the interaction picture for the time-dependent system. We then solve the corresponding interaction picture Schrodinger equation to leading order in the interaction. As the perturbation theory for time-dependent unperturbed Hamiltonian is not a very common technique, we provide a short but self-contained account of it here. Suppose that $\mathrm{U}_0(-\infty, t)$ is the unitary time-evolution operator of a time-dependent Hamiltonian $H_0(t)$. Then, we may define an interaction-picture wavefunction $|\Psi(t)\rangle_I$ related to the system wavefunction $|\Psi(\mathrm{t})\rangle$ by the relation $|\Psi(\mathrm{t})\rangle = \mathrm{U}_0(-\infty, t)|\Psi(t)\rangle_I$. Given a Hamiltonian $H = H_0(t) + V(t)$, it immediately follows that the Schrodinger equation for the wavefunction in the interaction picture is

$$i\hbar \frac{\partial}{\partial t}|\Psi(t)\rangle_I = V_I(t)|\Psi(t)\rangle_I,$$

with $V_I(t) = \mathrm{U}_0^\dagger(t)V(t)\mathrm{U}_0(t)$ (for brevity, we have omitted the notation of the initial time in the time-evolution operator). The general solution of this equation is prescribed by the time-ordered exponential as [5]:

$$|\Psi(t)\rangle_I = \mathrm{T}\exp\left[\frac{1}{i\hbar}\int_{-\infty}^{t} d\tau\ V_I(\tau)\right]|\Psi(-\infty)\rangle.$$

Thus far, the considerations have remained general. Now, we shall apply it to the case where the interaction Hamiltonian is between the driven electronic system and the quantized radiation field in vacuum. The corresponding unperturbed Hamiltonian is (from the previous section):

$$H_0(t) = H_{\mathrm{TDSE}} + H_{\mathrm{F}}\,,$$

where the "TDSE" Hamiltonian contains only the interaction between a quantum electronic system and the classical field, e.g., $\frac{1}{2m}\left(\mathbf{p} - q\mathbf{A}_{\mathrm{c}}(t)\right)^2 + U$. The corresponding interaction Hamiltonian, as derived in the previous section is:

$$V(t) = -\frac{q}{2m}\left(\left(\mathbf{p} - q\mathbf{A}_{\mathrm{c}}(t)\right)\cdot\mathbf{A}_{\mathrm{q}} + \mathbf{A}_{\mathrm{q}}\cdot\left(\mathbf{p} - q\mathbf{A}_{\mathrm{c}}(t)\right)\right) = -\frac{q}{m}\mathbf{A}_{\mathrm{q}}\cdot\left(\mathbf{p} - q\mathbf{A}_{\mathrm{c}}(t)\right).$$

The last equality follows from the fact that in free-space, the quantized radiation field is divergence-less, and thus commutes with the momentum operator. As the unperturbed Hamiltonian is separable in the electronic and photonic degrees of freedom, $\mathrm{U}_0(t)$ takes the form $\mathrm{U}_0(t) = \mathrm{U}_{\mathrm{TDSE}}(t)\mathrm{U}_{\mathrm{F}}(t) = \mathrm{U}_{\mathrm{TDSE}}(t)e^{-it\sum_{\mathbf{k}\sigma}\omega_k a^\dagger_{\mathbf{k}\sigma}a_{\mathbf{k}\sigma}}$. Defining for convenience $\mathbf{P}(t) = \mathbf{p} - q\mathbf{A}_{\mathrm{c}}(t)$, it follows that

$$i\hbar\frac{\partial}{\partial t}|\Psi(t)\rangle_I = \frac{q}{m}\mathrm{U}^\dagger_{\mathrm{TDSE}}(t)\mathbf{A}_{\mathrm{q}I}(\boldsymbol{r},t)\cdot\mathbf{P}(t)\mathrm{U}_{\mathrm{TDSE}}(t)|\Psi(t)\rangle_I,$$

using

$$\mathbf{A}_{\mathrm{q}I}(\mathbf{r},t) = \mathrm{U}^\dagger_{\mathrm{F}}(t)\mathbf{A}_{\mathrm{q}}(\mathbf{r})\mathrm{U}_{\mathrm{F}}(t) = \sum_{\mathbf{k}\sigma}\sqrt{\frac{\hbar}{2\varepsilon_0 Vck}}\left[\mathbf{e}_\sigma a_{k\sigma}\mathrm{e}^{i\mathbf{k}\cdot\mathbf{r}-i\omega_k t} + \mathbf{e}^*_\sigma a^\dagger_{\mathbf{k}\sigma}e^{-i\mathbf{k}\cdot\mathbf{r}+i\omega_k t}\right].$$

Up until now, the considerations have remained exact, independently of the strength of V. However, we shall now consider what happens when $V(t)$ is weak, so that its effect on the wavefunction is accurately accounted for by expanding the time-ordered exponential to first order in $V(t)$. Expanding the time-ordered exponential to first-order in $V(t)$ yields that the time-dependent wavefunction in the interaction picture is given by

$$|\Psi(t)\rangle_I = \left(1 + \frac{q}{i\hbar m}\int_{-\infty}^{t}\mathrm{U}^\dagger_{\mathrm{TDSE}}(\tau)\mathbf{A}_{\mathrm{q}I}(\mathbf{r},\tau)\cdot\mathbf{P}(\tau)\mathrm{U}_{\mathrm{TDSE}}(\tau)d\tau\right)|\Psi(-\infty)\rangle.$$

The most common case for the initial state is that in which the electron is in some eigenstate $|\phi_{\mathrm{i}}\rangle$ of $H_{\mathrm{atom}} = \frac{\mathbf{p}^2}{2m} + U$, as the external driving laser is off at $t = -\infty$. Meanwhile, the emitted electromagnetic field (which is also the entire field after the coherent shift from the previous section) is in the vacuum state $|0\rangle$. Thus, the time-dependent state, expanding the vector potential operator, is given explicitly by

$$|\tilde{\Psi}(t)\rangle_I = |\phi_{\mathrm{i}}\rangle|0\rangle + \frac{i\,q}{\hbar\,m}\sum_{\mathbf{k}\sigma}\sqrt{\frac{\hbar}{2\varepsilon_0 Vck}}\left[\left[\int_{-\infty}^{t}\mathrm{U}^\dagger_{\mathrm{TDSE}}(\tau)\left(\mathrm{e}^{-i\mathbf{k}\cdot\mathbf{r}}\mathbf{P}(\tau)\cdot\mathbf{e}^*_\sigma\right)\mathrm{e}^{i\omega_k\tau}d\tau\,\mathrm{U}_{\mathrm{TDSE}}(t)\right]|\phi_{\mathrm{i}}\rangle|\mathbf{k}\sigma\rangle,$$

with $|\mathbf{k}\sigma\rangle = a^\dagger_{\mathbf{k}\sigma}|0\rangle$ being a Fock state in which one photon is in the mode $|\mathbf{k}\sigma\rangle$. The tilde serves as a reminder that this wavefunction can also be transformed back by a displacement-operator to include the quantum state of the driving laser in the photonic state in a full QED Hamiltonian.

To retrieve the results of the main text, we may express the action of $\mathbf{P}(t)$ on $|\phi_{\mathrm{i}}\rangle$ by inserting a complete set of electronic states $\{|\phi_j\rangle\}$. It is usually convenient to choose these states to be the eigenstates of $H_{\mathrm{atom}} = \frac{\mathbf{p}^2}{2m} + U$, when the external driving laser is off at $t = -\infty$. It then follows that

$$\left|\widetilde{\Psi}(t)\right\rangle_I = |\phi_{\mathrm{i}}\rangle|0\rangle + \frac{i}{\hbar}\frac{q}{m}\sum_j \left|\phi_j\right\rangle \sum_{\mathbf{k}\sigma} \sqrt{\frac{\hbar}{2\varepsilon_0 Vck}} \left[\int_{-\infty}^{t} \left(\mathbf{P}_{j\mathrm{i}}(\tau)\cdot \mathbf{e}_\sigma^*\right) \mathrm{e}^{i\omega_k \tau} d\tau\right] |\mathbf{k}\sigma\rangle,$$

with $\mathbf{P}_{j\mathrm{i}}(t) = \left\langle \phi_{\mathrm{i}}(t)\right| \mathrm{e}^{-i\mathbf{k}\cdot\boldsymbol{r}} \mathbf{P}(t) \left|\phi_j(t)\right\rangle$, where $|\phi_{\mathrm{i}}(t)\rangle$ and $\left\{\left|\phi_j(t)\right\rangle\right\}$ are time-dependent states, which are connected with eigenstates $|\phi_{\mathrm{i}}(t)\rangle$ and $\left\{\left|\phi_j(t)\right\rangle\right\}$ by the following expressions:

$$|\phi_i(t)\rangle = \mathrm{U}_{\mathrm{TDSE}}(t)|\phi_{\mathrm{i}}\rangle \text{ and } \left\{\left|\phi_j(t)\right\rangle\right\} = \left\{\mathrm{U}_{\mathrm{TDSE}}(t)\left|\phi_j\right\rangle\right\} \text{ for all } j.$$

The wavefunction in the Schrodinger picture is immediately determined by applying the unitary time-evolution operator

$$\left|\widetilde{\Psi}(t)\right\rangle = |\phi_{\mathrm{i}}(t)\rangle|0\rangle + \frac{i}{\hbar}\frac{q}{m}\sum_j \left|\phi_j(t)\right\rangle \sum_{\mathbf{k}\sigma} \mathrm{e}^{-i\omega_k t} \sqrt{\frac{\hbar}{2\varepsilon_0 Vck}} \left[\int_{-\infty}^{t} d\tau \left(\mathbf{P}_{j\mathrm{i}}(\tau)\cdot \mathbf{e}_\sigma^*\right) \mathrm{e}^{i\omega_k \tau}\right] |\mathbf{k}\sigma\rangle. \quad (7)$$

The full wavefunction with the entire photonic field as part of the quantum state can be obtained by applying the displacement operator Supplementary Eq. (3), such that $|\Psi(t)\rangle = D(t)\left|\widetilde{\Psi}(t)\right\rangle$. However, we keep using the displaced wavefunction $\left|\widetilde{\Psi}(t)\right\rangle$ in both in the Supplementary Information and paper because it separates the electronic system and the emitted field from the driving field.

The time-dependent wavefunction enables immediate calculation of observables. Of particular interest in problems of strong-field-induced light, emission is the spectrum of photon emission, which is related to the expectation value of the energy of the electromagnetic field at long times $\varepsilon_{\mathrm{tot}}$:

$$\varepsilon_{\mathrm{tot}} = \sum_{\mathbf{k}\sigma} \hbar\omega_k \left\langle \widetilde{\Psi}(+\infty)\right| D^\dagger(t) a_{\mathbf{k}\sigma}^\dagger a_{\mathbf{k}\sigma} D(t) \left|\widetilde{\Psi}(+\infty)\right\rangle,$$

$$\varepsilon_{\mathrm{tot}} = \sum_{\mathbf{k}\sigma}\left(\hbar\omega_k|\alpha_{\mathbf{k}\sigma}|^2 + 2\mathrm{Re}\left[i\alpha_{\mathbf{k}\sigma}^* \frac{q}{m}\sqrt{\frac{\hbar\omega_k}{2\varepsilon_0 V}}\left[\int_{-\infty}^{t} d\tau \left(\mathbf{P}_{\mathrm{ii}}(\tau)\cdot\mathbf{e}_\sigma^*\right)\mathrm{e}^{i\omega_k\tau}\right]\right] + \sum_j \frac{q^2}{2m^2\varepsilon_0 V}\left|\int_{-\infty}^{+\infty}\left(\mathbf{P}_{j\mathrm{i}}(\tau)\cdot\mathbf{e}_\sigma^*\right)\mathrm{e}^{i\omega_k\tau} d\tau\right|^2\right).$$

$\varepsilon_{\mathrm{tot}}$ is the total energy of the driving and emitted fields. The energy of emitted field ε is expressed by the last term:

$$\varepsilon = \sum_{\mathbf{k}\sigma}\sum_j \frac{q^2}{2m^2\varepsilon_0 V}\left|\int_{-\infty}^{+\infty}\left(\mathbf{P}_{j\mathrm{i}}(\tau)\cdot\mathbf{e}_\sigma^*\right)\mathrm{e}^{i\omega_k\tau} d\tau\right|^2.$$

The discrete summation over all possible states, in the limit of an infinitely large box, can be expressed as an integration:

$$\sum_{\mathbf{k}} \rightarrow \frac{V}{(2\pi)^3 c^3} \int d\Omega \, d\omega \, \omega^2.$$

Thus, the emitted energy per unit frequency and per unit solid angle is given by:

$$\frac{d\varepsilon}{d\omega d\Omega} = \sum_{j,\sigma} \frac{q^2\omega^2}{16\pi^3 \varepsilon_0 m^2 c^3} \left| \int_{-\infty}^{+\infty} \left(\mathbf{P}_{j\mathrm{i}}(\tau) \cdot \mathbf{e}_\sigma^*\right) \mathrm{e}^{i\omega\tau} d\tau \right|^2, \quad (8)$$

where $\sum_j$ is a summation over all electron states $|\phi_j(t)\rangle$, and $\sum_\sigma$ is a summation over all possible photon polarizations.

In terms of a practical calculation of the spectrum of Eq. (S8), one should abide by the following "algorithm". (1) Choose a complete, orthonormal basis of wavefunctions for the electron $\{|\phi_j\rangle\}$ at time $t_i = -\infty$. It is convenient to choose the basis $\{|\phi_j\rangle\}$ in such way that the initial electronic state $|\phi_\mathrm{i}\rangle$ is part of this basis. Due to the unitarity of the time-evolution operator for the electron, $\mathrm{U}_{\mathrm{TDSE}}(t)$, this basis stays complete and orthonormal for all times, because a unitary operator is reversible and preserves the norm and the scalar product. Thus, each basis vector $\{|\phi_j\rangle\}$ evolves in time $|\phi_j(t)\rangle = \mathrm{U}_{\mathrm{TDSE}}(t)|\phi_j\rangle$. Note that this yields a *time-dependent* basis whose vectors are *not* connected to energy eigenvalues (because there are no energy eigenvalues for a general time-dependent Hamiltonian). (2) Calculate this time-dependent basis, for example, by solving the time-dependent Schrodinger equation governed by $H_{\mathrm{TDSE}}(t)$. (3) Given this time-dependent basis, calculate matrix elements of the form $\mathbf{P}_{j\mathrm{i}}(t) = \langle \phi_j(t) | \mathrm{e}^{-i\mathbf{k}\cdot\mathbf{r}}(\mathbf{p} - q\mathbf{A}_\mathrm{c}) | \phi_\mathrm{i}(t) \rangle$. From here, the spectrum is now fully specified.

Note that the considerations here have thus far made no reference to high-harmonic generation. This prescription can be used for *any* problem where a driving field leads to emission of light at new frequencies (or even for spontaneous emission without a driving field). Thus, this formalism, which could be called *strong-field quantum electrodynamics* (SFQED), can be used to address many problems in quantum nonlinear optics.

Supplementary Note 3: Dipole approximation for the driving field and the emitted field

In general, the computation of the momentum matrix elements $\mathbf{P}_{j\mathrm{i}}(t) = \langle \phi_\mathrm{i}(t) | \mathrm{e}^{-i\mathbf{k}\cdot\mathbf{r}} \mathbf{P}(t) | \phi_j(t) \rangle$ involves the integration of an electromagnetic plane wave over the time-dependent wavefunctions. The computation of these time-dependent wavefunctions also involves accounting for the spatial variation of the driving field over the electronic system. The spatial variations of the driving field are governed by the wavelength of light at visible/infrared frequencies, which is much larger than any characteristic scale of spatial variation of the wavefunction of the driven electronic system. In

that case, spatial variations of the driving field can be neglected. This assumption is the dipole approximation for the driving field, which is applied in most simulations of the time-dependent Schrodinger equation.

Considering the Hamiltonian for an electron in a classical time-dependent field $H_{\mathrm{TDSE}} = \frac{1}{2m}\left(\mathbf{p} - q\mathbf{A}_c(\mathbf{r},t)\right)^2 + U$, the dipole approximation for the driving field simply amounts to the approximation $\mathbf{A}_\mathrm{c}(\boldsymbol{r},t) \approx \mathbf{A}_\mathrm{c}(t)$. Under this approximation, it is common to perform a unitary transformation on the Hamiltonian to describe the electron-field coupling in terms of the dipole-moment operator, rather than the momentum operator. As a reminder of this unitary transformation (see [1] for more details), the matter wavefunction $|\phi(t)\rangle$ is expressed as:

$$|\phi(t)\rangle = \exp\left[i\frac{q}{\hbar}\mathbf{A}_\mathrm{c}(t)\cdot\mathbf{r}\right]|\phi'(t)\rangle. \tag{9}$$

$|\phi'(t)\rangle$ then satisfies the following Schrodinger equation:

$$\left[\frac{\mathbf{p}^2}{2m} + U(\mathbf{r}) - q\mathbf{E}(t)\cdot\mathbf{r}\right]|\phi'(t)\rangle = i\hbar\frac{\partial}{\partial t}|\phi'(t)\rangle, \tag{10}$$

where the electric field $\mathbf{E}(t) = -\dot{\mathbf{A}}(t)$. In Supplementary Note 10, we quantify corrections beyond this dipole (also called long-wavelength) approximation for the driving field.

Now, we move to discuss the dipole approximation for the emitted harmonics. Often, the wavelength of the emitted light is much longer than the spatial scale at which the electronic wavefunction varies. In that case, the matrix elements are highly simplified by neglecting the spatial changes $\mathbf{P}_{j\mathrm{i}}(t) = \langle\phi_\mathrm{i}(t)|e^{-i\mathbf{k}\cdot\mathbf{r}}\mathbf{P}(t)|\phi_j(t)\rangle \approx \langle\phi_\mathrm{i}(t)|e^{-i\mathbf{k}\cdot\mathbf{r}_0}\mathbf{P}(t)|\phi_j(t)\rangle$, with $\mathbf{r}_0$ the location of the nucleus of the atom, taken below as the origin without loss of generality. This dipole approximation avoids the need to integrate a plane wave over the electron wavefunction.

Additionally, under this dipole approximation for the emitted fields, the matrix elements may also be expressed in terms of a dipole $\mathbf{d}_{j\mathrm{i}}(t) = \langle\phi_j(t)|q\mathbf{r}|\phi_i(t)\rangle$, rather than the momentum operator:

$$\mathbf{P}_{j\mathrm{i}}(t) \approx \langle\phi_j(t)|(\mathbf{p} - q\mathbf{A}_\mathrm{c})|\phi_\mathrm{i}(t)\rangle = \frac{im}{\hbar}\langle\phi_j(t)|[H_{\mathrm{TDSE}},\mathbf{r}]|\phi_\mathrm{i}(t)\rangle = \frac{m}{q}\frac{\partial}{\partial t}\mathbf{d}_{j\mathrm{i}}(t),$$

$$\Rightarrow \int_{-\infty}^{t} d\tau\, \mathrm{e}^{i\omega_k\tau}\frac{\partial}{\partial\tau}\mathbf{d}_{j\mathrm{i}}(\tau) = \mathrm{e}^{i\omega_k\tau}\mathbf{d}_{j\mathrm{i}}(\tau)\Big|_{-\infty}^{t} - i\omega_k\int_{-\infty}^{t} d\tau\, \mathrm{e}^{i\omega_k\tau}\mathbf{d}_{j\mathrm{i}}(\tau) \approx -i\omega_k\int_{-\infty}^{t} d\tau\, \mathrm{e}^{i\omega_k\tau}\mathbf{d}_{j\mathrm{i}}(\tau),$$

$$\Rightarrow \int_{-\infty}^{t} d\tau\, \mathrm{e}^{i\omega\tau}\mathbf{P}_{j\mathrm{i}}(\tau) = -\frac{im\omega_k}{q}\int_{-\infty}^{t} d\tau\, e^{i\omega_k\tau}\mathbf{d}_{j\mathrm{i}}(\tau).$$

Under the dipole approximation for the emitted field Supplementary Eqs. (7) and (8) transform into:

$$|\tilde{\Psi}(t)\rangle = \left(|\phi_{\mathrm{i}}(t)\rangle|0\rangle + \sum_j |\phi_j(t)\rangle \frac{1}{\hbar}\sum_{\mathbf{k}\sigma} \mathrm{e}^{-i\omega_k t}\sqrt{\frac{\hbar\omega_k}{2\varepsilon_0 V}}\left[\int_{-\infty}^{t}\left(\mathbf{d}_{j\mathrm{i}}(\tau)\cdot\mathbf{e}_\sigma\right)\mathrm{e}^{i\omega_k\tau}d\tau\right]|\mathbf{k}\sigma\rangle\right), \tag{11}$$

$$\frac{d\varepsilon}{d\omega d\Omega} = \sum_{j,\sigma}\frac{q^2\omega^4}{2(2\pi)^3\varepsilon_0 c^3}\left|\int_{-\infty}^{+\infty}\left(\mathbf{d}_{j\mathrm{i}}(\tau)\cdot\mathbf{e}_\sigma^*\right)\mathrm{e}^{i\omega\tau}d\tau\right|^2.$$

After the integration over solid angle and summation of the polarization, we have the following expression for the emission per unit frequency:

$$\frac{d\varepsilon}{d\omega} = \sum_j \frac{\omega^4}{6\pi^2\varepsilon_0 c^3}\left|\int_{-\infty}^{+\infty}\mathbf{d}_{j\mathrm{i}}(t)\mathrm{e}^{i\omega t}dt\right|^2 \tag{12}$$

Supplementary Eqs. (11) and (12) correspond to Eqs. (3) and (4) of the main text. The dipole approximation for the emitted field gives accurate results in many cases, however, in the next sections we will show that it can break down, specifically when considering high-harmonic generation at very high harmonics.

We can apply for Supplementary Eq. (12) to the case of spontaneous emission in time-*independent* electronic systems by changing the time-dependent states $|\phi_i(t)\rangle$ and $|\phi_j(t)\rangle$ to the initial eigenvectors $|\phi_i\rangle$ and $|\phi_j\rangle$. Substituting these eigenvectors in the dipole matrix element and integrating over the frequency, we can get the formula for the rate of spontaneous emission Γ_s:

$$\Gamma_s = \frac{\omega^3}{3\pi\varepsilon_0\hbar c^3}\left|\mathbf{d}_{ji}\right|^2, \tag{13}$$

where i and j are the initial and final state of the electronic system. Supplementary Eq. (13) coincides with the well-known result for spontaneous emission in free-space [1]. Besides Supplementary Eq. (13) representing a sanity check on our derivations, the manner of derivation also suggests that we may interpret Supplementary Eq. (12) in the following way: the process of HHG emission is very similar to spontaneous emission, with the "only" difference being that electron states are dressed by the strong external field $\mathbf{A}_{\mathrm{c}}(t)$.

Supplementary Note 4: The single-atom and the many-atom regimes of HHG.

This section discusses the differences between HHG for a single atom and HHG for many atoms. Although we use the dipole approximation, the same arguments also apply to the expressions without the dipole approximation. We found Supplementary Eq. (12) for the radiation emission, however, the conventional result differs from Supplementary Eq. (12) and reads [3]:

$$\frac{d\varepsilon}{d\omega} = \frac{\omega^4}{6\pi^2\varepsilon_0 c^3}\left|\int_{-\infty}^{+\infty}\mathbf{d}_{\mathrm{ii}}(t)\mathrm{e}^{i\omega t}dt\right|^2, \tag{14}$$

where $\mathbf{d}_{\text{ii}} = \langle \phi_{\text{i}}(t)|q\mathbf{r}|\phi_{\text{i}}(t)\rangle$ is the (*diagonal*) time-dependent matrix element. It can be shown (see discussion in [6]) that in the case of many-atoms emitting HHG, the formula for the emission is the following:

$$\frac{d\varepsilon}{d\omega} = N^2 \cdot \frac{\omega^4}{6\pi^2\varepsilon_0 c^3}\left|\int_{-\infty}^{+\infty} \mathbf{d}_{\text{ii}}(t)e^{i\omega t}dt\right|^2 + N \cdot \sum_{j\neq \text{i}} \frac{\omega^4}{6\pi^2\varepsilon_0 c^3}\left|\int_{-\infty}^{+\infty} \mathbf{d}_{j\text{i}}(t)\text{e}^{i\omega t}dt\right|^2, \quad (15)$$

where N is the number of atoms. Supplementary Eq. (15) gives Supplementary Eq. (13) in the case of emission by a single atom (single-atom regime). However, if we have many atoms ($N \gg 1$), the contribution of the "incoherent" part (i.e., the part in Supplementary Eq. (15) proportional to N) gradually becomes negligible and we get Supplementary Eq. (14) multiplied by N^2. Hence, we have two quite distinct regimes of HHG: a single-atom regime described by Supplementary Eq. (13), and a many-atom regime described by Supplementary Eq. (14).

Consequently, the single-atom regime has major conceptual differences from the many-atom regime. For example, the energy difference between different states $|\phi_j(-\infty)\rangle$ before the interaction leads to emission spectra that contain shifted frequency combs (the shifts correspond to transition frequencies of the atom in the absence of the driving field). We show examples of such spectra in the main text. We should note that these conceptual differences arise only from the quantum nature of the HHG emission and we do not consider phase-matching effects here.

Supplementary Note 5: Non-perturbative calculation of HHG for a "one-level" model

In the previous sections, we have calculated the (quantum) emission of light by a single strongly driven atom. In those calculations, we assumed that the coupling between the driven atom and the *quantized* field is weak and that the effect of this coupling could be considered to first order in perturbation theory. For a single atom, as we shall show below, this is generally valid. However, for the case of many atoms (as in a gaseous target), this is not always the case, as the number of photons produced can be much larger than one (even if each individual atom produces much less than one photon). In that case, higher orders in perturbation theory can become important. Here we provide a non-perturbative account of the photon emission.

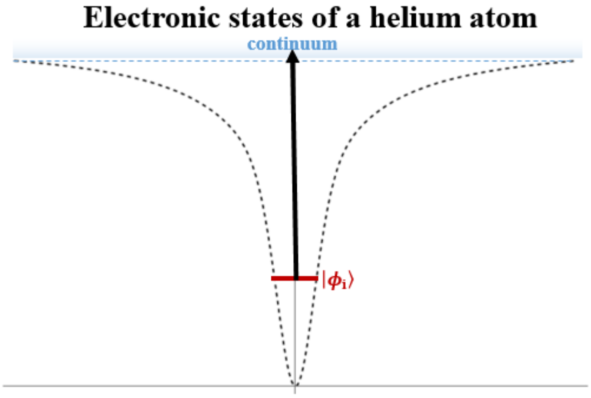

Supplementary Figure 1. Illustration of a "one-level" model approximation of a helium atom

In this section, we will use the "one-level" model, which takes into account only transitions between the initial ground state of the atom and the continuum (i.e. ionization) as it depicted in Supplementary Figure 1. Only the transitions ending in the same state as the initial state need to be retained in the many-atom regime. When transitions to other electronic states are negligible, the "one-level" model gives a correct prediction of the emission spectrum. This situation is often referred to as the non-depleted ground state approximation [2] and is often the case for conventional parameters in HHG.

In cases that can be described by the "one-level" model, the photon statistics is trivial (Poissonian statistics), as we show below. In this model, we have a single time-dependent level established by applying the driving field to the initial state of the atom. Accordingly, the time-dependent state of the system may be written as $|\widetilde{\Psi}(t)\rangle = |\phi_{\mathrm{i}}(t)\rangle|\chi(t)\rangle$, with $|\chi(t)\rangle$ some photonic wavefunction. The resulting time-dependent Schrodinger equation for the interaction picture photonic wavefunction can then be approximated as:

$$i\hbar\frac{\partial}{\partial t}|\chi(t)\rangle_I = -\sum_{\mathbf{k}\sigma}\sqrt{\frac{\hbar}{2\varepsilon_0 Vck}}\left[a_{\mathbf{k}\sigma}\mathrm{e}^{-i\omega_k t}\frac{\partial}{\partial t}(\mathbf{d}_{\mathrm{ii}}(t)\cdot\mathbf{e}_\sigma) + a^\dagger_{\mathbf{k}\sigma}\mathrm{e}^{i\omega_k t}\frac{\partial}{\partial t}(\mathbf{d}_{\mathrm{ii}}(t)\cdot\mathbf{e}^*_\sigma)\right]|\chi(t)\rangle_I,$$

where $|\chi(t)\rangle = \mathrm{U_F}|\chi(t)\rangle_I$.

The solution of this equation may immediately be written as [7]:

$$|\chi(t)\rangle_I = \prod_{\mathbf{k}\sigma}\exp[\beta_{\mathbf{k}\sigma}a^\dagger_{\mathbf{k}\sigma} - \beta^*_{\mathbf{k}\sigma}\mathrm{a}_{\mathbf{k}\sigma}]\,|\chi(-\infty)\rangle,$$

where $|\chi(-\infty)\rangle = |0\rangle$ is the initial photonic wavefunction (taken to be the vacuum state) and

$$\beta_{\mathbf{k}\sigma} = \sqrt{\frac{\omega_k}{2\varepsilon_0\hbar V}}\int_{-\infty}^{t}\mathrm{e}^{i\omega_k\tau}(\mathbf{d}_{\mathrm{ii}}(\tau)\cdot\mathbf{e}_\sigma)\,d\tau.$$

Therefore $|\widetilde{\Psi}(t)\rangle$ has the following form:

$$|\widetilde{\Psi}(t)\rangle = |\phi_i(t)\rangle|\chi(t)\rangle = |\phi_i(t)\rangle\left(\mathrm{U_F}(t)\prod_{\mathbf{k}\sigma}\exp\left[\beta_{\mathbf{k}\sigma}a^\dagger_{\mathbf{k}\sigma} - \beta^*_{\mathbf{k}\sigma}a_{\mathbf{k}\sigma}\right]\right)|0\rangle.$$

Finally, we have:

$$|\widetilde{\Psi}(t)\rangle = |\phi_\mathrm{i}(t)\rangle \prod_{\mathbf{k}\sigma}\left|\beta_{\mathbf{k}\sigma}\mathrm{e}^{-i\omega_k t}\right\rangle. \quad (16)$$

Let us expand Supplementary Eq. (16) in a Taylor series up to the second order in the coupling $\beta_{\mathbf{k}\sigma}$:

$$|\widetilde{\Psi}(t)\rangle \approx |\phi_\mathrm{i}(t)\rangle\left(|0\rangle + \mathrm{e}^{-i\omega_k t}\sum_{\mathbf{k}\sigma}\beta_{\mathbf{k}\sigma}|\mathbf{k}\sigma\rangle + \sum_{\mathbf{k}_1\sigma_1\neq\mathbf{k}_2\sigma_2} e^{-i(\omega_{k_1}+\omega_{k_2})t}\beta_{\mathbf{k}_1\sigma_1}\beta_{\mathbf{k}_2\sigma_2}|\mathbf{k}_1\sigma_1\rangle|\mathbf{k}_2\sigma_2\rangle\right).$$

Note that the first two terms coincide with the part of Supplementary Eq. (11) coming from diagonal matrix elements, showing consistency with our previous results. From the last equation, we see that first-order perturbation theory is correct when we can neglect the last term, i.e., when the norm of the third term is much smaller than the norm of the second term:

$$\sum_{\mathbf{k}\sigma}|\beta_{\mathbf{k}\sigma}|^2 \ll 1.$$

This condition holds true in typical conditions for a single atom [3], hence in the single-atom regime, the first-order perturbation theory that we used before is well-justified. Importantly, in typical HHG experiments, the number of photons emitted is $N^2\sum_{\mathbf{k}\sigma}|\beta_{\mathbf{k}\sigma}|^2$ (where N is number of atoms), which can be much greater than one when N is very large. Hence, in the many-atom case, we cannot always use first-order perturbation theory and should derive the equations in a non-perturbative way, as is done in this section and the next section.

Going back to the initial reference frame, where the laser mode is populated with many photons, the corresponding wavefunction $|\Psi(t)\rangle$ is given by:

$$|\Psi(t)\rangle = \prod_{\mathbf{k}\sigma}\exp\left[i\,\mathrm{Im}\left[\alpha_{\mathbf{k}\sigma}\beta^*_{\mathbf{k}\sigma}(t)\mathrm{e}^{-i\omega_k t}\right]\right]|\phi_\mathrm{i}(t)\rangle\left|\left(\alpha_{\mathbf{k}\sigma}+\beta_{\mathbf{k}\sigma}(t)\right)\mathrm{e}^{-i\omega_k t}\right\rangle, \quad (17)$$

where $\left|\left(\alpha_{\mathbf{k}\sigma}+\beta_{\mathbf{k}\sigma}(t)\right)\mathrm{e}^{-i\omega_k t}\right\rangle$ is a coherent state in which the initial laser coherent state parameters $\alpha_{\mathbf{k}\sigma}$ have been displaced by the emitted field contribution $\beta_{\mathbf{k}\sigma}(t)$. Note that in the case of many atoms, $\beta_{\mathbf{k}\sigma}(t)$ should be changed to $N\beta_{\mathbf{k}\sigma}(t)$ in Supplementary Eq. (17). At harmonics of the driving laser, or any mode $\mathbf{k}\sigma$ that was empty before the emission, $\alpha_{\mathbf{k}\sigma} = 0$ and so $\beta_{\mathbf{k}\sigma}(t)$ dominates the mode.

Consequently, the HHG radiation is a tensor product of coherent states describing each harmonic frequency. A single coherent state emission of photons is described by a Poisson distribution, and this is also the type of radiation emitted by a classical current [7]. Specifically, Poissonian statistics gives zero Mandel parameter $Q = 0$ and zero squeezing η. These parameters are defined by the following formulas: the Mandel parameter is $Q = \frac{\langle n^2\rangle - \langle n\rangle^2}{\langle n\rangle} = 1$, where n is the photon number operator; the squeezing $\eta = 10|\log|4\Delta X^2||$ [2], where ΔX^2 is the variance of the quadrature operator As we will see in the next chapter, all the quantum features arises from taking into account transitions between different levels of an atom.

Supplementary Note 6: Non-perturbative calculation of the quantum state of the emitted HHG

In the previous section, we considered a "one-level" model and came to the conclusion that only transitions between different states give non-trivial photon statistics. In this section, we take into account not only transitions between ground state $|\phi_1\rangle$ and continuum, but also the transitions between different levels of the atom (Supplementary Figure 2)

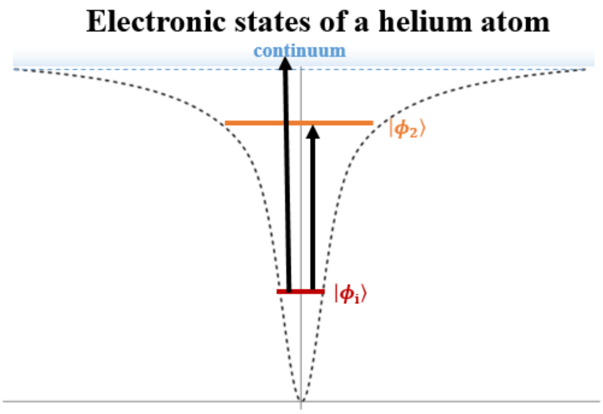

Supplementary Figure 2. Illustration of electronic states in a helium atom

We present the Schrodinger equation in the interaction picture from Supplementary Note 2:

$$i\hbar\frac{\partial}{\partial t}|\Psi(t)\rangle_I = V_I(t)|\Psi(t)\rangle_I,$$

with $V_I(t) = \mathrm{U}_0^\dagger(t)V(t)\mathrm{U}_0(t)$ (for brevity, we have omitted the notation of the initial time in the time-evolution operator). The $V_I(t)$ term in the dipole approximation can be decomposed in the basis of the stationary atomic states $\{|\phi_m\rangle\}$:

$$V_I(t) = -\sum_{m,n} \mathbf{A}_{qI}(t) \cdot \dot{\mathbf{d}}_{mn}(t) \cdot |\phi_m\rangle\langle\phi_n|,$$

$$\mathbf{A}_{qI}(\mathbf{r},t) = \sum_{\mathbf{k}\sigma} \sqrt{\frac{\hbar}{2\varepsilon_0 V\omega_k}} \mathbf{e}_\sigma \left[a_{\mathbf{k}\sigma}\mathrm{e}^{-i\omega_k t} + a_{\mathbf{k}\sigma}^\dagger \mathrm{e}^{i\omega_k t}\right].$$

The general solution of the Schrodinger equation $|\Psi(\mathrm{t})\rangle_I$ can also be presented on the basis of stationary atomic states:

$$|\Psi(t)\rangle_I = \sum_m |\chi^m\rangle|\phi_m\rangle,$$

where $|\chi^m\rangle$ is the photonic state corresponding to the mth state of the atom (note that the normalization condition requires $\sum_m \langle\chi^m|\chi^m\rangle = 1$). Substituting this form of the general wavefunction into the Schrodinger equation, we get:

$$i\hbar \frac{\partial|\chi^m\rangle}{\partial t} = -\sum_{k\sigma} \sqrt{\frac{\hbar}{2\varepsilon_0 V\omega_k}} \left[a_{\mathbf{k}\sigma}e^{-i\omega_k t} + a_{\mathbf{k}\sigma}^\dagger e^{i\omega_k t}\right] \sum_n \left(\mathbf{e}_\sigma \cdot \dot{\mathbf{d}}_{mn}(t)\right)|\chi^n\rangle.$$

To proceed, we make the assumption that each frequency can be treated independently (equivalent to neglecting the entanglement between different harmonics). Then, for each harmonic, we can write the following equation:

$$i\hbar \frac{\partial|\chi_{\mathbf{k}\sigma}^m\rangle}{\partial t} = -\sqrt{\frac{\hbar}{2\varepsilon_0 V\omega_k}} \left[a_{\mathbf{k}\sigma}\mathrm{e}^{-i\omega_k t} + a_{\mathbf{k}\sigma}^\dagger \mathrm{e}^{i\omega_k t}\right] \sum_n \left(\mathbf{e}_\sigma \cdot \dot{\mathbf{d}}_{mn}(t)\right)|\chi_{\mathbf{k}\sigma}^n\rangle.$$

We can rewrite this equation in a matrix form. We build the matrix $\dot{\mathbf{D}}(t)$ of $\dot{\mathbf{d}}_{mn}(t)$ elements: $\mathbf{D}(t) = \{\mathbf{d}\}_{mn}$ and define the column of photonic states:

$$|\mathrm{X}_{\mathbf{k}\sigma}\rangle = \begin{pmatrix} |\chi_{\mathbf{k}\sigma}^1\rangle \\ |\chi_{\mathbf{k}\sigma}^2\rangle \\ \cdots \end{pmatrix}.$$

In this matrix form, we have the following time-dependent equation for the photonic states:

$$\frac{\partial|\mathrm{X}_{\mathbf{k}\sigma}\rangle}{\partial t} = i\sqrt{\frac{\omega_k\omega_0}{6\pi^2\varepsilon_0 c^3}}\,\alpha\left[a_{\mathbf{k}\sigma}\mathrm{e}^{-i\omega_k t} + a_{\mathbf{k}\sigma}^\dagger \mathrm{e}^{i\omega_k t}\right]\dot{D}(t)|\mathrm{X}_{\mathbf{k}\sigma}\rangle, \tag{18}$$

where $\alpha = \sqrt{\frac{3\pi^2 c^3}{V\omega_k^2\omega_0}}\cos\theta_{d\sigma}$ relates to the amplitude of the emitted harmonic, ω_0 is the driving frequency, and $\theta_{d\sigma}$ is the angle between the dipole moment and the photon's polarization. For the numerical calculations below we use the initial condition $|\chi_{\mathbf{k}\sigma}^n(t_\mathrm{i})\rangle = \delta_{1n}|0\rangle$, where δ_{1n} is the Kronecker delta.

We solve (Supplementary Eq. 18) numerically for different frequencies ω_k. For the example presented in Figure 3 and 4 in the main text, we take into the account only the ground and the first excited state of the atom and neglect the other transitions. Such simplification is justified by Figure 2 in the main text because we can see that the largest contribution for the emission is connected with the ground $|\phi_1\rangle \equiv |\mathrm{g}\rangle$ and the 1st excited state $|\phi_2\rangle \equiv |\mathrm{e}\rangle$. Under such an assumption, we have the following simplifications:

$$|\mathrm{X}_{\mathbf{k}\sigma}(t_\mathrm{i})\rangle = \begin{pmatrix} |0\rangle \\ 0 \end{pmatrix},$$

$$|\mathrm{X}_{\mathbf{k}\sigma}(t)\rangle = \begin{pmatrix} |\chi_{\mathbf{k}\sigma}^{\mathrm{g}}(t)\rangle \\ |\chi_{\mathbf{k}\sigma}^{\mathrm{e}}(t)\rangle \end{pmatrix},$$

$$D(t) = \begin{pmatrix} d_{\mathrm{gg}}(t) & d_{\mathrm{ge}}(t) \\ d_{\mathrm{eg}}(t) & d_{\mathrm{ee}}(t) \end{pmatrix},$$

$$|\Psi(t)\rangle_I = |\chi^{\mathrm{g}}(t)\rangle|\mathrm{g}\rangle + |\chi^{\mathrm{e}}(t)\rangle|\mathrm{e}\rangle.$$

We developed a numerical simulation that finds $|\chi^{\mathrm{g}}(t)\rangle$ and $|\chi^{\mathrm{e}}(t)\rangle$ for each frequency. In the previous sections, we distinguished between two different regimes of emission: the single-atom regime and the many-atom regime. For the single atom regime, the wavefunction is given by the expression above for $|\Psi(t)\rangle_I$. However, for the many-atom regime, due to the incoherent nature of the transitions between different states, the part of the state projected on $|\mathrm{g}\rangle$ contributes dominantly to the emission $\left|\Psi_{\mathrm{many}}(t)\right\rangle_I \approx |\chi^{\mathrm{g}}(t)\rangle|\mathrm{g}\rangle$.

For both the single- and many-atom regimes, the quantum statistics of the emitted light can be calculated using $|\Psi_N(\mathrm{t})\rangle_I$ by tracing-out the atomic degree of freedom. As examples, we calculate the Mandel parameter Q and the squeezing parameter η [7].

$$Q = \frac{\langle n^2\rangle - \langle n\rangle^2}{\langle n\rangle} - 1,$$

$$\eta = 10|\log[4(\langle X^2\rangle - \langle X\rangle^2)]|,$$

using the following definitions $n = a^\dagger a$ and $X = \frac{1}{2}(a + a^\dagger)$. The final formulas for each operator $\hat{O}$ (e.g., n, n^2, X, X^2) after tracing-out the atomic degree of freedom, have the following form:

$$\langle \hat{O} \rangle = \langle \chi^{\mathrm{g}} | \hat{O} | \chi^{\mathrm{g}} \rangle + \langle \chi^{\mathrm{e}} | \hat{O} | \chi^{\mathrm{e}} \rangle.$$

For the many-atom regime, we can neglect the terms that contain $|\chi^{\mathrm{e}}\rangle$.

The numerical simulations depend on the parameter α that relates to the number of atoms and the duration of the driving pulse. Below, we estimate the parameter α. Using Supplementary Eq. 12 from Supplementary Note 2, the average number of photons per unit frequency is given by:

$$\frac{d\langle n_{\omega_k} \rangle}{d\omega} = \sum_j \frac{\omega_k^3}{6\pi^2 \varepsilon_0 \hbar c^3} \left| \int_{-\infty}^{+\infty} \mathbf{d}_{j\mathrm{i}}(t) \mathrm{e}^{i\omega_k t} dt \right|^2.$$

To estimate α, we compare this result with the 1st order perturbation theory solution of Supplementary Eq. 12, which gives:

$$\langle n_{\omega_k} \rangle \approx \sum_j \frac{\omega_k^3}{6\pi^2 \varepsilon_0 \hbar c^3} \omega_0 \; |\alpha|^2 \left| \int_{-\infty}^{+\infty} \mathbf{d}_{j\mathrm{i}}(t) \mathrm{e}^{i\omega_k t} dt \right|^2.$$

Comparing these formulas, we conclude that $|\alpha|^2 \sim \frac{\Delta\omega}{\omega_0}$. Here, $\Delta\omega$ is the spectral width of the harmonic that directly relates to the duration of the driver pulse T as $\Delta\omega \sim \frac{1}{T}$. For the many-atom regime, the final expression for α also takes into account the number of atoms N_{p} (that are phase-matched in their emission). The result of this estimate yields $\alpha \approx \alpha_0 \cdot N_{\mathrm{p}}$, where $\alpha_0 = \frac{1}{\sqrt{N_{\mathrm{c}}}}$ and N_{c} is the number of cycles in the driving pulse. We have $N_{\mathrm{c}} = 40$ (Figure 2 in the main text) and thus form a single atom $\alpha_0 \approx 0.16$. Note that through this rough estimate we can see that the emission efficiency and dependence on the intensity of the driver field do not strongly influence α (they still alter the emitted number of photons).

For the numerical simulation, we take α in the case of a single atom equals to $\alpha = \alpha_0 \approx 0.16$. In the many-atom regime, we use $\alpha = 0.158 \cdot N_{\mathrm{p}}$ for different values of a phase-matched number of atoms $N_{\mathrm{p}} = 10^3, N_{\mathrm{p}} = 10^4$ and $N_{\mathrm{p}} = 5 \cdot 10^5$. We should note that the phase-matched number of atoms can be many times smaller than the real number of atoms ($N_{\mathrm{p}} \ll N$). Hence, to change the phase-matched number of atoms N_{p}, it is necessary to change the density of atoms in the gas (e.g., by changing pressure).

All the results of numerical simulations of Supplementary Eq. 18 with $\alpha_0 \approx 0.16$ are presented and discussed in the manuscript in Figures 3 and 4. Specifically, Figure 4 in the main text presents

results for both Q and η for three different values of N_{p}. These examples show that given a fixed emission spectrum, the stronger the emission, the more squeezing we get. Nevertheless, even in the limit of the weakest emission – in the case of a single atom – other quantum features appear.

Supplementary Note 7: Photonic states in the single-atom and the many-atom regimes

This section discusses the quantum optical nature of the emitted photon in HHG and general emission processes in nonlinear optics driven by a strong field. We recall the formula for the electron+field wavefunction (Supplementary Eq. (11)) in the single-atom regime:

$$|\tilde{\Psi}(t)\rangle = |\phi_{\mathrm{i}}(t)\rangle|0\rangle + \sum_{j}|\phi_{j}(t)\rangle\frac{1}{\hbar}\sum_{\boldsymbol{k}\sigma}\mathrm{e}^{-i\omega_{k}t}\sqrt{\frac{\hbar\omega_{k}}{2\varepsilon_{0}V}}\left[\int_{-\infty}^{t}d\tau\,\mathrm{e}^{i\omega_{k}\tau}\big(\boldsymbol{d}_{j\mathrm{i}}(\tau)\cdot\mathbf{e}_{\sigma}\big)\right]|\mathbf{k}\sigma\rangle.$$

Perhaps one of the most interesting features of this wavefunction is the *entanglement* between the photonic state and the electronic state, in the sense that we cannot decompose the wavefunction into a tensor product of a photonic state and an atomic state. We can write the photon states as

$$|1_{\mathrm{HHG},j\mathrm{i}}\rangle = \frac{1}{\hbar}\sum_{\mathbf{k}\sigma}\mathrm{e}^{-i\omega_{k}t}\sqrt{\frac{\hbar\omega_{k}}{2\varepsilon_{0}V}}\left[\int_{-\infty}^{t}d\tau\,\mathrm{e}^{i\omega_{k}\tau}\big(\mathbf{d}_{j\mathrm{i}}(\tau)\cdot\mathbf{e}_{\sigma}\big)\right]|\mathbf{k}\sigma\rangle,$$

and write the combined quantum state as $|\tilde{\Psi}(t)\rangle = |\phi_{\mathrm{i}}(t)\rangle|0\rangle + \sum_{j}|\phi_{j}(t)\rangle|1_{\mathrm{HHG},j\mathrm{i}}\rangle$.

Entanglement, of course, is a purely quantum effect and has many consequences; many of which are beyond the scope of this work. One thing that we will comment on is that the entanglement appears to be “ruined” in the many-atom regime because only the initial state of the atom gives a considerable contribution to the wavefunction:

$$|\tilde{\Psi}(t)\rangle = |\phi_{\mathrm{i}}(t)\rangle\big(|0\rangle + |1_{\mathrm{HHG,ii}}\rangle\big), \tag{19}$$

where $|\tilde{\Psi}(t)\rangle$ is normalized on the number of atoms N in the many-atom regime. From Supplementary Eq. (19) we can see that the combined wavefunction is a tensor product of a photonic state and an atomic state, hence there is no entanglement.

Let us investigate the final photonic state in Supplementary Eq. (19):

$$|\Psi_{\mathrm{ph}}(t)\rangle = |0\rangle + |1_{\mathrm{HHG,ii}}\rangle. \tag{20}$$

We see that the photonic state $|1_{\mathrm{HHG,ii}}\rangle$ is the superposition of different frequencies. Supplementary Eq. (20) tells us that each photon in the HHG carries all the frequencies of the entire spectrum because it contains matrix element $\mathbf{d}_{\mathrm{ii}}(\tau)$. This prediction can be tested experimentally by measuring the field autocorrelation function. In the manuscript, we propose how such an experiment might look. Here, we derive the field autocorrelation function [4]:

$$A^{(2)}(\tau) = \int_{-\infty}^{+\infty} \mathbf{E}^{(-)}(t+\tau)\cdot\mathbf{E}^{(+)}(t)d\tau, \tag{21}$$

where $A^{(2)}(\tau)$ is the autocorrelation, $\mathbf{E}^{(-)}(t) = -i\sum_{\mathrm{k}\sigma}\sqrt{\frac{\hbar}{2\varepsilon_0 Vck}}(k\mathbf{e}_\sigma)a_{\mathbf{k}\sigma}^{\dagger}\mathrm{e}^{-i(\mathbf{k}\cdot\mathbf{r}-\omega_k t)}$ and $\mathbf{E}^{(+)}(t) = i\sum_{\mathbf{k}\sigma}\sqrt{\frac{\hbar}{2\varepsilon_0 Vck}}(k\mathbf{e}_\sigma)a_{\mathbf{k}\sigma}e^{i(\mathbf{k}\cdot\mathbf{r}-\omega_k t)}$.

We define $\mathbf{r}_{\mathrm{ed}}$ as the spatial vector connecting the detector and the emission location (Fig. S3).

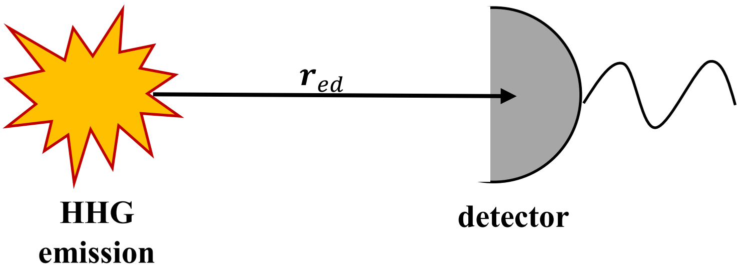

Supplementary Figure 3. The distance between the emitter and detector is $r_{\mathrm{ed}} = |\mathbf{r}_{\mathrm{ed}}|$.

We substitute the fields and get:

$$A^{(2)}(\tau) = \sum_{\mathbf{k}\sigma}\sum_{\mathbf{k}'\sigma'} \mathrm{e}^{-i\omega_{k'}\tau}\sqrt{\frac{\hbar\omega_{k'}}{2\varepsilon_0 V}}\sqrt{\frac{\hbar\omega_k}{2\varepsilon_0 V}}(\mathbf{e}_\sigma\cdot\mathbf{e}_{\sigma'})a_{\mathbf{k}\sigma}^{\dagger}a_{\mathbf{k}'\sigma'}e^{-i(\mathbf{k}-\mathbf{k}')\cdot\mathbf{r}_{\mathrm{ed}}}\,2\pi\delta(\omega_k-\omega_{k'}).$$

We then transform the discrete sum in the integral to $\sum_{\mathbf{k}} \to \int \frac{V}{(2\pi)^3c^3}\omega^2 d\omega d\Omega$:

$$A^{(2)}(\tau) = \sum_{\sigma,\sigma'}\int d\Omega\int d\Omega'\int d\omega_k\frac{\hbar\omega_k^5}{2\varepsilon_0(2\pi)^5c^6}\,\mathrm{e}^{i\omega_k\tau}V(\mathbf{e}_\sigma\cdot\mathbf{e}_{\sigma'})\mathrm{e}^{-ik(\mathbf{e}_{\mathrm{k}}-\mathbf{e}_{\mathrm{k}'})\cdot\mathbf{r}_{\mathrm{ed}}}a_{\mathbf{k}\sigma}^{\dagger}a_{\mathbf{k}\sigma'}.$$

Therefore, the autocorrelation function equals:

$$\langle A^{(2)}(\tau)\rangle = \langle\Psi_{\mathrm{ph}}|A^{(2)}(\tau)|\Psi_{\mathrm{ph}}\rangle =$$

$$= \sum_{\sigma,\sigma'}\int d\Omega\int d\Omega'\int d\omega_k\frac{\hbar\omega_k^5}{2\varepsilon_0(2\pi)^5c^6}\,\mathrm{e}^{-i\omega_k\tau}V(\mathbf{e}_\sigma\cdot\mathbf{e}_{\sigma'})e^{-ik(\mathbf{e}_{\mathrm{k}}-\mathbf{e}_{\mathrm{k}'})\cdot\mathbf{r}_{\mathrm{ed}}}\langle\Psi_{\mathrm{ph}}|a_{\mathbf{k}\sigma}^{\dagger}a_{\mathbf{k}\sigma'}|\Psi_{\mathrm{ph}}\rangle,$$

where

$$\langle \Psi_{\rm ph} | a_{\mathbf{k}\sigma}^{\dagger} a_{\mathbf{k}\sigma'} | \Psi_{\rm ph} \rangle = \frac{\omega_k}{2\varepsilon_0 \hbar V} \int_{-\infty}^{+\infty} d\tau\, \mathrm{e}^{i\omega_k \tau} (\mathbf{d}_{\rm ii}(\tau) \cdot \boldsymbol{e}_{\sigma}) \int_{-\infty}^{+\infty} d\tau\, \mathrm{e}^{-i\omega_k \tau} (\mathbf{d}_{\rm ii}^{*}(\tau) \cdot \mathbf{e}_{\sigma'}).$$

After integration over solid angles, we get the final expression for the field autocorrelation:

$$\langle A^{(2)}(\tau) \rangle \approx \int \frac{\omega_k^6}{8\pi^3 \varepsilon_0^2 c^6} \left(\frac{\sin k r_{ed}}{k r_{ed}} \right)^2 \left[\left(\left| \int_{-\infty}^{+\infty} d\tau\, \mathrm{e}^{i\omega_k \tau} \left(\mathbf{d}_{\rm ii}(\tau) - \frac{\mathbf{r}_{\rm ed}}{r_{ed}} \cdot \mathbf{d}_{\rm ii}(\tau) \right) \right|^2 \right) \right] \mathrm{e}^{i\omega_k \tau} \, d\omega. \quad (22)$$

Here $\langle A^{(2)}(\tau) \rangle$ is calculated in the approximation of $k r_{ed} \gg 1$, up to the first order in $\frac{1}{k r_{ed}}$.

Supplementary Note 8: The quiver radius as an important characteristic of the system

In this section, we provide an estimate for the magnitude of the "dipole" moment associated with high-harmonic generation. The dipole moment can be estimated as the classical dipole moment of the atom during the HHG process (associated with the orbit of the ionized electron around the parent nucleus). For an intense driving field, one can assume that the electron is free and define the so-called "quiver radius" a, which is the amplitude of motion of a free electron in the electric field of the laser $E = E_0 \cos \omega_0 t$ with frequency ω_0.

$$a = \frac{q E_0}{m \omega_0^2},$$

where m is mass of the electron. In this case, qa can be used as an estimate of the magnitude of the dipole moment. The quiver radius can also be expressed in terms of the intensity and wavelength of driving field as:

$$a = \frac{q \lambda_0^2}{4\pi^2 m c^2} \sqrt{\frac{2I}{c \varepsilon_0}}, \quad (23)$$

where I is the intensity of the driving field and λ_0 is the wavelength of the driving field. In the main text, we take as characteristic values $I = 2 \cdot 10^{14}\ \mathrm{W \cdot cm^{-2}}$ and $\lambda_0 = 800$ nm. In this case, the quiver radius is roughly 1 nm.

Supplementary Note 9: The magnitude of non-dipolar effects in both the driving and the emitted fields in HHG.

The dipole approximation for the driving field is accurate when the spatial size of electron orbit (e.g., the quiver radius, a) is much smaller than the wavelength of the driving field:

$$a \ll \lambda_0. \tag{24}$$

This holds true in all experiments so far because beyond-dipole corrections scale with the typical ratio $\frac{2\pi}{\lambda_0} a \sim 10^{-2}$ (for $\lambda_0 = 800$ nm and $a = 1$ nm).

What is more interesting is the validity of the dipole approximation for the emitted field, which holds provided that a is much smaller than the wavelength λ of the emitted light. This approximation should be violated for the higher harmonics that can have wavelengths on the few-nanometer scale. In that case, the emission power scales with the parameter $\frac{2\pi}{\lambda} a = n \cdot \frac{2\pi}{\lambda_0} a$, where n is the harmonic number. For instance, this parameter for 101th harmonic equals to $101 \cdot \frac{2\pi}{\lambda_0} a \sim 1$. In this case, one strongly expects multipolar contributions to become relevant.

The general formula for the emission beyond the dipole approximation for the emitted field is Supplementary Eq. (8). However, Supplementary Eq. (8) can be hard to calculate numerically. We make simplifications in the next two sections. We consider only the diagonal element $\mathbf{P}_{\text{ii}}$ inline with our discussion of many-atom effects. Phase-matching effects can be added on top of the calculation, as we discuss at the end of the section. We have the following formula for the emission beyond the dipole approximation (corresponds to Eq. (6) of the main text):

$$\frac{d\varepsilon}{d\omega d\Omega} = \sum_\sigma \frac{q^2}{2(2\pi)^3 \varepsilon_0} \frac{\omega^2}{m^2 c^3} \left| \int_{-\infty}^{+\infty} (\mathbf{P}_{\text{ii}}(t) \cdot \mathbf{e}_\sigma^*) e^{i\omega t} dt \right|^2, \tag{25}$$

where $\mathbf{P}_{\text{ii}}(t) = \langle \phi_{\text{i}}(t) | e^{-i\mathbf{k}\cdot\mathbf{r}} \left(\mathbf{p} - \frac{q}{c}\mathbf{A}_{\text{c}}(t)\right) | \phi_{\text{i}}(t) \rangle$.

It proves useful to write $\mathbf{P}_{\text{ii}}(t) \cdot \mathbf{e}_\sigma^*$ in an equivalent form:

$$\mathbf{P}_{\text{ii}}(t) \cdot \mathbf{e}_\sigma^* = \frac{1}{2} \left\langle \phi_{\text{i}}(t) \middle| \left\{ \left(\mathbf{p} - \frac{q}{c}\mathbf{A}_{\text{c}}(t)\right), e^{-i\mathbf{k}\cdot\mathbf{r}} \right\} \middle| \phi_{\text{i}}(t) \right\rangle,$$

where $\left\{ \left(\mathbf{p} - \frac{q}{c}\mathbf{A}_{\text{c}}(t)\right), e^{-i\mathbf{k}\cdot\mathbf{r}} \right\} = e^{-i\mathbf{k}\cdot\mathbf{r}} \left(\mathbf{p} - \frac{q}{c}\mathbf{A}_{\text{c}}(t)\right) + \left(\mathbf{p} - \frac{q}{c}\mathbf{A}_{\text{c}}(t)\right) e^{-i\mathbf{k}\cdot\mathbf{r}}$. This form is equivalent to the previous because the commutator $\left[\left(\mathbf{p} - \frac{q}{c}\mathbf{A}_{\text{c}}(t)\right) \cdot \mathbf{e}_\sigma^*, e^{-i\mathbf{k}\cdot\mathbf{r}} \right] = 0$.

We reintroduce the operator $\mathbf{P}(t) = \mathbf{p} - q\mathbf{A}_{\text{c}}(t)$ and calculate the emission spectrum:

$$\frac{d\varepsilon}{d\omega d\Omega}=\frac{q^2}{2(2\pi)^3\varepsilon_0}\frac{\omega^2}{m^2c^3}\int_{-\infty}^{+\infty}d\tau_1\,\mathrm{e}^{i\omega\tau_1}\int_{-\infty}^{+\infty}d\tau_2\,\mathrm{e}^{-i\omega\tau_2}\times$$

$$\times\frac{1}{2}\left\{\int dV_1\int dV_2\,\phi^*(\mathbf{r_2},\tau_2)\phi^*(\mathbf{r_1},\tau_1)\sum_\sigma(\mathbf{P}_1(\tau_1)\cdot\mathbf{e}^*_\sigma)\left(\mathbf{P}_2^\dagger(\tau_2)\cdot\mathbf{e}_\sigma\right)\phi(\mathbf{r}_1,\tau_1)\phi(\mathbf{r}_2,\tau_2),\mathrm{e}^{-ik\mathbf{e_k}\cdot(\mathbf{r_1}-\mathbf{r_2})}\right\},$$

Using the identity

$$\sum_\sigma(\mathbf{P}_1(\tau_1)\cdot\mathbf{e}^*_\sigma)(\mathbf{P}_2^\dagger(\tau_2)\cdot\mathbf{e}_\sigma)=\left(\mathbf{P}_1(\tau_1)\cdot\mathbf{P}_2^\dagger(\tau_2)\right)-(\mathbf{P}_1(\tau_1)\cdot\mathbf{e_k})(\mathbf{P}_2^\dagger(\tau_2)\cdot\mathbf{e_k}).$$

Here $\mathbf{P}_1$ acts on coordinate $\mathbf{r}_1$ and $\mathbf{P}_2$ on coordinate $\mathbf{r}_2$. Let us assume that we have a driving field linear polarized along the z direction. Then, the z polarization components of all the expectation values are much larger than the components in the other directions because the largest electron's displacement is in the direction of the driving field $\mathbf{E}(t)$. Hence, $\langle\phi_\mathrm{i}(t)|\mathbf{r}|\phi_\mathrm{i}(t)\rangle\approx\boldsymbol{e_z}\langle\phi_\mathrm{i}(t)|z|\phi_\mathrm{i}(t)\rangle$ and $\langle\phi_\mathrm{i}(t)|\mathbf{P}|\phi_\mathrm{i}(t)\rangle\approx\mathbf{e_z}\langle\phi_\mathrm{i}(t)|P_z|\phi_\mathrm{i}(t)\rangle$.

Under such an approximation, we get:

$$\frac{d\varepsilon}{d\omega d\Omega}\approx\frac{q^2}{2(2\pi)^3\varepsilon_0}\frac{\omega^2\sin^2\theta}{m^2c^3}\int_{-\infty}^{+\infty}d\tau_1\,\mathrm{e}^{i\omega\tau_1}\int_{-\infty}^{+\infty}d\tau_2\,\mathrm{e}^{-i\omega\tau_2}\times$$

$$\times\frac{1}{2}\left\{\int dV_1\int dV_2\,\phi^*(\mathbf{r_2},\tau_2)\phi^*(\mathbf{r_1},\tau_1)\left((P_1)_{z_1}(\tau_1)(P_2)_{z2}(\tau_2)\right)\phi(\mathbf{r_1},\tau_1)\phi(\mathbf{r_2},\tau_2),\mathrm{e}^{-ik(z_1-z_2)\cos\theta}\right\}=$$

$$=\frac{q^2\omega^2\sin^2\theta}{2(2\pi)^3\varepsilon_0m^2c^3}\int_{-\infty}^{+\infty}d\tau_1\,\mathrm{e}^{i\omega\tau_1}\int_{-\infty}^{+\infty}d\tau_2\,\mathrm{e}^{-i\omega\tau_2}\sum_n\frac{(ik)^n\cos^n\theta}{n!}\sum_{m=0}^{n}\frac{(-1)^m n!\,\langle 1/2\{P_z,z^m\}\rangle\langle 1/2\{P_z,z^{n-m}\}\rangle}{(n-m)!\,m!}.$$

It can be proven that the following equation holds true:

$$\frac{1}{2}\{P_z(t),z^{n-1}\}=\frac{im}{\hbar}\frac{1}{n}[H_\mathrm{TDSE},z^n],$$

where P_z has the following properties: $P_z(t)=\frac{im}{\hbar}[H_\mathrm{TDSE},z]$ and $[P_z,z]=-i\hbar$. Hence:

$$\frac{d\varepsilon}{d\omega d\Omega}=\frac{1}{2(2\pi)^3\varepsilon_0}\frac{\omega^4\sin^2\theta}{c^3}\int_{-\infty}^{+\infty}d\tau_1\,\mathrm{e}^{i\omega\tau_1}\int_{-\infty}^{+\infty}d\tau_2\,\mathrm{e}^{-i\omega\tau_2}\sum_n\left(\frac{ik}{q}\right)^n\cos^n\theta\sum_{m=0}^{n}\frac{(-1)^m\langle d^{m+1}(\tau_1)\rangle\langle d^{n-m+1}(\tau_2)\rangle}{(n-m)!\,m!}.$$

Finally, we get:

$$\frac{d\varepsilon}{d\omega d\Omega}=\frac{\omega^4\sin^2\theta}{2(2\pi)^3\varepsilon_0c^3}\sum_n\left(\frac{ik}{q}\right)^n\cos^n\theta\sum_{m=0}^{n}\frac{(-1)^m\langle d^{m+1}(\omega)\rangle\langle d^{n-m+1}(\omega)\rangle^*}{(n-m+1)!(m+1)!},\qquad(26)$$

where $\langle d^m(\omega)\rangle=\int\mathrm{e}^{i\omega\tau}\langle\phi_\mathrm{i}(\tau)|(qz)^m|\phi_\mathrm{i}(\tau)\rangle d\tau$ and $\langle d^m(\omega)\rangle^*=\int\mathrm{e}^{-i\omega\tau}\langle\phi_\mathrm{i}(\tau)|(qz)^m|\phi_\mathrm{i}(\tau)\rangle d\tau$.

Supplementary Eq. (26) gives all the multipolar contributions to the emission spectrum. In the main text we presented the first three terms in the expansion:

$$\frac{d\varepsilon}{d\omega d\Omega} \approx \frac{\omega^4 \sin^2\theta}{2(2\pi)^3\varepsilon_0 c^3}\left(|\langle d(\omega)\rangle|^2 + \frac{k}{q}\cos\theta \ \mathrm{Im}[\langle d(\omega)\rangle\langle d^2(\omega)\rangle^*] + \frac{k^2}{q^2}\cos^2\theta\left(\frac{1}{4}|\langle d^2(\omega)\rangle|^2 - \frac{1}{3}\mathrm{Re}[\langle d(\omega)\rangle\langle d^3(\omega)\rangle^*]\right)\right). \quad (27)$$

The first term here is the known dipolar term, equivalent to the emission spectrum under the dipole approximation for the emitted field. The third term (that scales like $\sim k^2/q^2$) is the quadrupole emission. The second term (that scales like $\sim k/q$) is the emission by an interaction between the dipolar and quadrupolar terms. After the integration over all angles, the interaction term cancels out and we have (equivalent to Eq. (7) in the main text):

$$\frac{d\varepsilon}{d\omega} = \frac{\omega^4}{6\pi^2\varepsilon_0 c^3}\left(|\langle d(\omega)\rangle|^2 + \frac{1}{5}\frac{\omega^2}{q^2c^2}\left(\frac{1}{4}|\langle d^2(\omega)\rangle|^2 - \frac{1}{3}\mathrm{Re}[\langle d(\omega)\rangle\langle d^3(\omega)\rangle^*]\right)\right). \quad (28)$$

Supplementary Eq. (28) contains the dipolar and the quadrupolar emission. In the main text, we show numerically that the quadrupolar term becomes significant in case of an intense enough driving field (typically $I > 10^{14}\ \mathrm{W \cdot cm^{-2}}$). Interestingly, the quadrupolar term contains even harmonics. As we discussed in the main text, the directionality of the quadrupolar emission cause it to diminish in regular phase-matching conditions. Therefore, the observation of beyond-dipole corrections requires an excitation from two or more directions to create new phase-matching conditions. Alternatively, experiments without phase-matching conditions (e.g., from thin areas of interaction as in some solid HHG) are also expected to show beyond-dipole corrections.

Supplementary Note 10: Coherent and incoherent parts of the radiation

In Supplementary Note 5, we briefly discussed the coherent and incoherent parts of HHG radiation and the difference between the single-atom and the many-atom regimes of emission. Here we will give a qualitative explanation for why some part of the radiation is incoherent. This result shows the potential of the quantum approach of SFQED and especially important in the many-atom regime of HHG.

Let us consider N atoms inside a small volume relative to the wavelength of the driving field, such that emission from the individual atoms will be in phase. We neglect the interactions between the atoms and only consider their interactions with the external laser pulse and with the vacuum electromagnetic field. For the sake of simplicity, we will consider a model of two-level atoms with ground $|\mathrm{g}\rangle$ and excited states $|\mathrm{e}\rangle$. These assumptions can be relaxed in a more general derivation which we will not attempt here.

We introduce the raising operator s^+, the lowering operator s^-, and the operator s^z, for each single atom:

$$s^-|\mathrm{e}\rangle = |\mathrm{g}\rangle, \qquad s^-|\mathrm{g}\rangle = 0,$$

$$s^+|\mathrm{e}\rangle = 0, \qquad s^+|\mathrm{e}\rangle = |\mathrm{g}\rangle,$$

$$s^z|\mathrm{e}\rangle = |\mathrm{e}\rangle, \qquad s^z|\mathrm{g}\rangle = -|\mathrm{g}\rangle,$$

We construct the collective operators for the many atoms:

$$S^+ = \sum_i s_i^+ , \qquad S^- = \sum_i s_i^- , \qquad S^z = \sum_i s_i^z,$$

where $\sum_i$ is a sum over all atoms.

Recalling the single-atom formula Supplementary Eq. (12), the emission from a two-level system model initially in the ground state has the following form:

$$\frac{d\varepsilon}{d\omega} = \frac{\omega^4}{6\pi^2\varepsilon_0 c^3}\left(\left|\mathbf{d}_{\mathrm{gg}}(\omega)\right|^2 + \left|\mathbf{d}_{\mathrm{eg}}(\omega)\right|^2\right),$$

where the initial condition on the atom was in the ground state $|g\rangle$. Moreover, $\mathbf{d}_{\mathrm{gg}}(\omega) = \int \langle \mathrm{g}(t)|\mathbf{d}|\mathrm{g}(t)\rangle \mathrm{e}^{i\omega t} dt$, and $\mathbf{d}_{\mathrm{eg}}(\omega) = \int \langle \mathrm{e}(t)|\mathbf{d}|\mathrm{g}(t)\rangle \mathrm{e}^{i\omega t} dt$, with $\mathbf{d}$ being the dipole moment operator for the two-level system. We can write the same formula in a different way:

$$\frac{d\varepsilon}{d\omega} = \frac{\omega^4}{6\pi^2\varepsilon_0 c^3}\langle \mathrm{g}|d^\dagger(\omega)d(\omega)|\mathrm{g}\rangle,$$

where $d(\omega) = \begin{pmatrix} d_{\mathrm{gg}}(\omega) & d_{\mathrm{ge}}(\omega) \\ d_{\mathrm{eg}}(\omega) & d_{\mathrm{ee}}(\omega) \end{pmatrix} = d_{\mathrm{ge}}s^- + d_{\mathrm{eg}}s^+ + d_{\mathrm{gg}}\left(\frac{1}{2} - s^z\right) + d_{\mathrm{ee}}\left(\frac{1}{2} + s^z\right).$

For an ensemble of atoms initially at the ground state, this formula can be generalized in analogy with [11] to yield:

$$\frac{d\varepsilon}{d\omega} = \frac{\omega^4}{6\pi^2\varepsilon_0 c^3}\langle \mathrm{g} \ldots \mathrm{g}|D^\dagger(\omega)D(\omega)|\mathrm{g} \ldots \mathrm{g}\rangle, \tag{30}$$

where $|\mathrm{g} \ldots \mathrm{g}\rangle$ is the ground state of the ensemble of atoms, $D(\omega)$ is the total dipole moment of the system:

$$D(\omega) = d_{\mathrm{ge}}S^- + d_{\mathrm{eg}}S^+ + d_{\mathrm{gg}}\left(\frac{1}{2} - S^z\right) + d_{\mathrm{ee}}\left(\frac{1}{2} + S^z\right). \tag{31}$$

which together with Supplementary Eq.(30) yields a general expression for the emission of N atoms.

This model is limited because it does not capture the dynamics of the atomic state; however, it is a useful approximation [11]. We use this result to make a qualitative prediction about the emission spectrum, substituting Supplementary Eq.(31) in Supplementary Eq.(30):

$$\frac{d\varepsilon}{d\omega} = \frac{\omega^4}{6\pi^2\varepsilon_0 c^3}\left(N^2\left|d_{\mathrm{gg}}(\omega)\right|^2 + N\left|d_{\mathrm{eg}}(\omega)\right|^2\right), \tag{32}$$

This formula Supplementary Eq. (32) coincides with Supplementary Eq. (15) and qualitatively explains why the radiation from the transition dipole d_{eg} is incoherent – it scales with N. The radiation from the expectation dipole of the dipole d_{gg} is coherent – it scales with N^2.

In this section, we showed the derivation of the coherent and the incoherent parts of the HHG emission for an ensemble of atoms. We again note that the many-body theory written here is just a qualitative approach because it does not take into account the dynamics of the atomic system. Moreover, the approach applied in this section does not capture the statistical properties of the emitted photonic field. Nevertheless, this section makes a first step in the construction of a consistent quantum many-body theory of HHG.

Supplementary Note 11: Comparison and proof of equivalence of two approaches for calculating the emission spectrum

In this section, we compare our formalism with the derivation in the paper [12], which calculates radiation emission from a bound electron in a strong field in a different approach than ours. According to Eq. (4) and Eq. (11) in this paper, we have the Schrodinger equation:

$$i\hbar\frac{\partial}{\partial t}|\Psi\rangle = (H_0(t) + V_{\mathrm{int}})|\Psi\rangle,$$

where H_0 is the Hamiltonian including the classical part of the field, and V_{int} is the perturbation connected with the quantum part of the field. In our notions, the interaction part looks like:

$$V_{\mathrm{int}} = \frac{q}{m}\mathbf{A}_{\mathbf{q}} \cdot \mathbf{P},$$

where in dipole approximation

$$\mathbf{A}_{\mathbf{q}}(r,t) \approx \sum_{\mathbf{k}\sigma}\sqrt{\frac{\hbar}{2\varepsilon_0 Vck}}\left[\mathbf{e}_\sigma a_{\mathbf{k}\sigma}\mathrm{e}^{-i\omega_k t} + \mathbf{e}_\sigma^* a_{\mathbf{k}\sigma}^\dagger(k)\mathrm{e}^{+i\omega_k t}\right] \text{ and } \mathbf{P}(t) = \mathbf{p} - q\mathbf{A}_{\mathrm{c}}(t).$$

According to Eq. (6) in [12], unperturbed state evolves by ordinary TDSE:

$$i\hbar\frac{\partial}{\partial t}|\psi_{\mathrm{i}}\rangle = H_0(t)|\psi_{\mathrm{i}}\rangle.$$

Then, the perturbed state can be found in the following form according to Eq. (9) and Eq. (1) in [12]:

$$|\Psi\rangle = |\psi_{\mathrm{i}}(t)\rangle \otimes |0\rangle + \sum_{k\sigma} |\psi_{\mathbf{k}\sigma}\rangle \otimes |\mathbf{k}\sigma\rangle.$$

In the 1st order, we can write the following equation for $|\psi_{\mathbf{k}\sigma}\rangle$ (Eq. 12):

$$i\hbar\frac{\partial}{\partial t}|\psi_{\mathbf{k}\sigma}\rangle = H_0(t)|\psi_{\mathbf{k}\sigma}\rangle + \frac{q}{m}\sqrt{\frac{\hbar}{2\varepsilon_0 Vck}}\left[\mathbf{e}_\sigma^* \cdot \mathbf{P}\mathrm{e}^{+i\omega_k t}\right]|\psi_i\rangle.$$

Hence, [12] suggests firstly to solve TDSE for $|\psi_{\mathrm{i}}\rangle$ (Eq. 6), then to solve TDSE *for each* **k** and σ for the perturbation $|\psi_{\mathbf{k}\sigma}\rangle$ (Eq. 12 in [12]) and then find the emission by the formula (Eq. 13 in [12]):

$$\varepsilon = \sum_{\mathbf{k}\sigma} \hbar\omega\langle\psi_{\mathbf{k}\sigma}|\psi_{\mathbf{k}\sigma}\rangle.$$

In comparison, our approach is to solve TDSE $i\hbar\frac{\partial}{\partial t}|\psi(t)\rangle = H_0(t)|\psi(t)\rangle$ not only for the initial state but also for all orthogonal states, thus we form a full orthogonal basis $\{|\psi_j(t)\rangle\}$. To prove the equivalence of the two approaches, we decompose the state $|\psi_{\mathbf{k}\sigma}\rangle$ in time-dependent basis:

$$|\psi_{\mathbf{k}\sigma}\rangle = \sum_j C_j(t)|\psi_j(t)\rangle,$$

with the equation for the $C_j(t)$ coefficients being the following:

$$i\hbar\frac{\partial}{\partial t}C_j(t) = \frac{q}{m}\sqrt{\frac{\hbar}{2\varepsilon_0 Vck}}\langle\psi_j|\mathbf{P}|\psi_i\rangle\mathbf{e}_\sigma^* e^{i\omega_k t}.$$

Solving this equation we get the following result:

$$|\psi_{\mathbf{k}\sigma}\rangle = \frac{q}{m\hbar i}\sqrt{\frac{\hbar}{2\varepsilon_0 Vck}}\sum_j \int d\tau\,(\mathbf{P}_{j\mathrm{i}} \cdot \mathbf{e}_\sigma^*)\mathrm{e}^{i\omega_k\tau}\,|\psi_j\rangle.$$

Then finally, using the equation for the emission (Eq. 13 in [12]) we get:

$$\varepsilon = \frac{q^2}{2m^2\varepsilon_0}\sum_j\sum_{\mathbf{k}\sigma}\frac{1}{V}\left|\int d\tau\,(\mathbf{P}_{j\mathrm{i}} \cdot \mathbf{e}_\sigma^*)\mathrm{e}^{i\omega_k\tau}\right|^2,$$

which is exactly Supplementary Eq. (8). Thus, both approaches are mathematically equivalent.

Supplementary References

[1] Cohen-Tannoudji C., Dupont-Roc J., Grynberg G., *Atom-Photon Interactions: Basic Processes and Applications,* (Wiley-VCH, New York, 1989).

[2] Jin, C. *Theory of Nonlinear Propagation of High Harmonics Generated in a Gaseous Medium*, (Springer, New York, 2013).

[3] Schultz T., Vrakking M., *Attosecond and XUV Physics: Ultrafast Dynamics and Spectroscopy*, (Wiley-VCH, Wienheim, 2014).

[4] Mandel L., Wolf E., *Optical Coherence and Quantum Optics*, (Cambridge University Press, New York, 1995).

[5] Peskin, M. E., Schroeder, D. V. *An Introduction to Quantum Field Theory*, (CRC Press, Boca Raton, 2018).

[6] Eberly J.H., Fedorov M. V., Spectrum of light scattered coherently or incoherently by a collection of atoms, *Phys. Rev. A* (**45**), 7 (1992).

[7] Scully M. O., Zubairy M. S., *Quantum Optics,* (Cambridge University Press, New York, 1997).

[8] Foldi P. et al., Quantum-optical nature for the dynamics of high-order-harmonic generation, *Phys. Rev. A* (**94**), 013853 (2016).

[9] Tsatrafyllis N., Kominis I. K., Gonoskov I. A. and Tzallas P. High-order harmonics measured by the photon statistics of the infrared driving-field exiting the atomic medium, *Nature Comm.* **8**, 15170 (2017).

[10] Gonoskov I. A. Tsatrafyllis N., Kominis I. K and Tzallas P. Quantum optical signatures in strong-field laser physics: Infrared photon counting in high-order harmonic generation, *Sci. Rep.* **6**, 32821 (2016).

[11] Gross M., Haroche S. Superradiance: an essay on the theory of collective spontaneous emission, *Physics Reports* **93**, 5, 301-396 (1982).

[12] Bogatskaya A. V., Volkova E. A. and Popov A. M, *Laser Phys. Lett.,* V.**14**, No. 5, 055301 (2017).
